# DMF-MALDI: droplet based microfluidic combined to MALDI-TOF for focused peptide detection

**DOI:** 10.1038/s41598-017-06660-8

**Published:** 2017-07-28

**Authors:** Kiarach Mesbah, Robert Thai, Sarah Bregant, Florent Malloggi

**Affiliations:** 1 LIONS, NIMBE, CEA, CNRS, Université Paris-Saclay, CEA Saclay 91191, Gif sur Yvette Cedex, France; 2 SIMOPRO, JOLIOT, DRF, CEA, Université Paris-Saclay, CEA Saclay 91191, Gif sur Yvette Cedex, France

## Abstract

We present an automated droplet microfluidic system (DMF) to generate monitored nanoliter aqueous droplets in oil and their deposition on a commercial stainless steel plate for MALDI-TOF analysis of peptides or protein digests. We demonstrate that DMF-MALDI combination focuses the analyte on the MALDI plate, increasing considerably the homogeneity of the dried material. This results in a 30times enhanced MALDI-TOF MS signal for a model peptide, allowing a significant improvement of the detection sensitivity limit (down to few tens of attomoles). Moreover, positive detection can be achieved from sub-nanomolar peptides solutions and better overall protein sequence coverages are obtained from few tens attomoles of protein digest. These results make DMF-MALDI a promising approach for the treatment of peptides samples as well as a key component for an integrated approach in the proteomic field.

## Introduction

Matrix-assisted laser desorption/ionization time-of-flight mass spectrometry (MALDI-TOF MS) is recognized as a powerful tool in biological studies, especially in biomarker discovery and in the proteomic field^1^. Like other mass spectrometry (MS)-based technique, it presents many advantages such as sensitivity, accuracy and speed, making this technique of considerable interest for biomarkers analysis^2–4^. The detection sensitivity of the MALDI-TOF technique is strongly correlated to the mass of the analyzed biomolecule (to 1 picomole for protein and 100 attomoles for peptide of choice). Many efforts - sample preparation protocols^5–7^, surface modified plate^8–10^, original MALDI target^11^ or electrowetting techniques^12^ - are still in progress to overcome shot to shot reproducibility and/or to enhance the detection sensitivity by MALDI-TOF. Most of them allow a more homogeneous crystallization over the spotted area by a focusing effect.

Droplet-based microfluidics (DMF) finds applications in many fields such as single-cell analysis^13^, digital PCR^14^, chemical synthesis^15^ or biomarker analysis^16^. To selectively analyze and quantify droplets content the efforts have been performed to adapt DMF to analytical detection techniques: microscopy with^17^ or without fluorescence^18^, Raman spectroscopy^19^, electrochemistry^18^ capillary electrophoresis^20^, or mass spectrometry^21^. Interfacing DMF with MS technology, in particular MALDI-TOF MS, is very promising in the field of clinical studies, especially in proteomics. Beside this potency only few developments have been reported in the literature. First proof of concept to integrate MALDI-TOF MS with droplet-based microfluidic devices was described by Hatakeyama *et al*.^22^ for the analysis of a nanomolar amount of a plant derived toxic substance. In this study, the matrix mixture is added on dried deposited analyte. Momotenko *et al*. combined microfluidics and MALDI-TOF MS detection to generate nanodroplets for chemical analysis of fingerprint contamination by picric acid with a limit of detection of 28.5 nanomoles^23^. This nanomole detection range is consistent with a non-oil droplets carrier (air plugs) that favors the loss of analyte by adsorption (biofouling). More recently, Dittrich’s group developed a novel interface to monitor the enzymatic conversion of Angiotensin I to Angiotensin II by ACE (Angiotensin Converting Enzyme)^24^. In their automatized interface, nanoliter aqueous droplets immersed in oil are created in a microfluidic T-junction and deposited on a home-made hydrophobic MALDI plate with 26000 hydrophilic areas. The increasing amount of Angiotensin II over time was followed by the deposition of more than 8000 single droplets. The assays have been performed with tens of femtomoles of Angiotensin peptides spotted on dried matrix. This detection range is intrinsically linked to the parameters of this enzymatic study implying substrate degradation by ACE. Overall in these studies, the detection threshold reached is in the nanomole or femtomole range. In other studies, attomolar sensitivity detection may be required to ensure the detection of some biomarkers at physiological level in biological fluids. Some challenging concerns in proteomics require the ability of dedicated developed analysis system to address the detection of low abundant proteins or peptides biomarkers from biological fluids. In this work, we present a droplet-based microsystem combined to MALDI-TOF to target peptide detection in the attomole range by fast, accurate and automatized spotting of droplets of matrix/analyte mixture. The device comprises a PDMS microchip in which few nL droplets are created before being automatically spotted on a commercial MALDI stainless steel plate. Thanks to this DMF system, we develop a spotting method for the analysis of peptides or protein (through protein digest) encapsulated in microdroplets which leads to a higher detection sensitivity by MALDI-TOF MS compared to conventional droplet deposition.

## Results and Discussion

**Figure 1:**
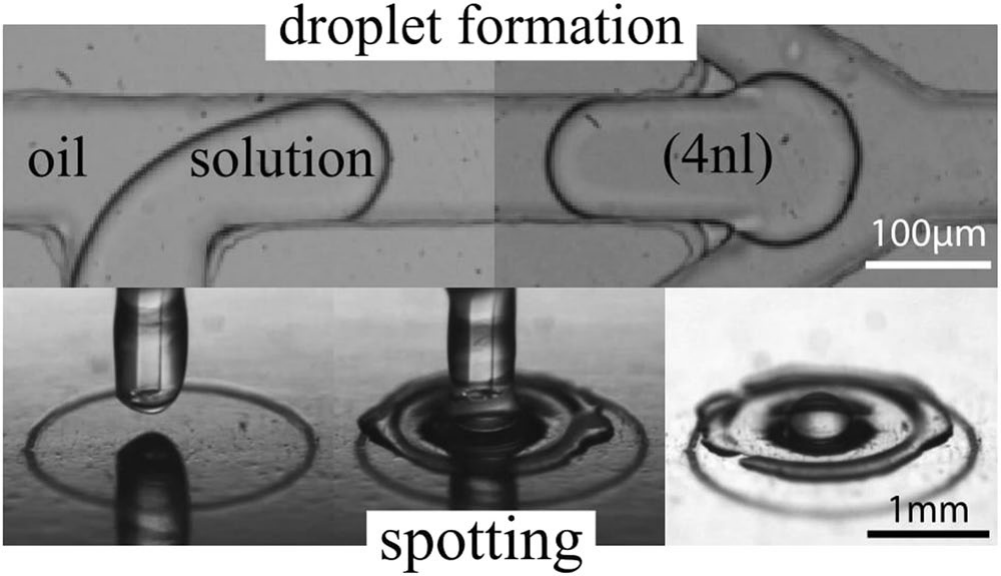
DMF-MALDI Set-up: droplet creation and spotting. Top: droplet generation (left), droplet spacing (right) within the PDMS microfluidic chip. Bottom: before deposition (left), during deposition (middle), after deposition (right) on stainless steel MALDI plate.

In droplet based microfluidics, the analyte is surrounded by an oil carrier that prevents any contact with the microchannel surfaces and hence the biofouling^25, 26^. For our microdevice, we selected hydrophobic PDMS in order to make water in oil droplets. Moreover, to enhance the oil wettability, the channels were coated with an hydrophobic solution and few percents of fluorinated alcohol were added in oil. Surfactants usually used to prevent droplets coalescence generate numerous peaks of interferences on the MALDI-TOF MS spectra. In our system, the droplets were thus generated free of surfactants using a T-junction and an additional oil entrance was engineered to space the droplets and prevent droplets coalescence (see Fig. 1-top). On plate, the spotted volume was controlled by the number of accumulated single droplet (4nL) at the outlet before moving to another plate position (see Fig. 1-bottom images). MALDI-TOF analysis requires co-crystallization of analyte sample including an organic matrix to achieve efficient desorption and ionization of the analyte. A one-step droplet deposition method was envisaged by creating droplets from a mixture of sample containing a model peptide (Ang II) including the classical HCCA matrix preparation acidified by 0.1% Trifluoroacetic Acid (TFA). First deposition experiments showed that the MALDI-TOF analysis of generated droplets gave drastically lower signal intensities for the target peak than the one obtained from standard deposition of the same sample. This was due to a lack of acidity preventing the protonation of matrix/Ang II mixture, one of the criteria required for the MALDI-TOF MS detection of Ang II. Indeed by replacing TFA in the matrix solution with other acids, the Ang II corresponding peak was detected with higher intensity in all acidic conditions, more specifically with trichloroacetic acid (TCA). This result points out that in first experiments, TFA may have migrated towards the oil phase during the droplet generation. We selected TCA at 1% in the HCCA matrix stock solution as the best composition for high sensitivity detection by MALDI-TOF MS.

**Figure 2:**
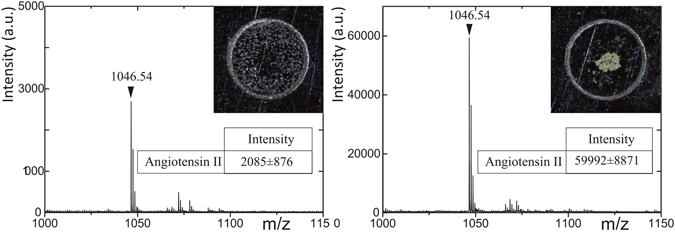
MALDI-TOF MS spectra of 5 femtomoles of Ang II. Left. Standard pipette deposition. Right. DMF deposition. Inserts: Dried mixture of peptide/matrix before laser desorption.

To estimate the gain of our platform for peptide detection, we applied the previously optimized parameters and compared our system with a conventional deposition procedure, i.e. manual pipetting. Same volumes (500 nL) containing Ang II mixed 1/1 (v/v) with acidified matrix (final concentration: 10 fmol/*μ*L) were spotted either by standard pipetting method or by accumulation of generated droplets by our system on the same commercial stainless steel MALDI plate (10 replicates). Figure 2 shows examples of MALDI-TOF MS spectra obtained in each case for a total amount of 5 femtomoles of spotted Ang II. A rapid look to the spot areas underlines that the sample solution drying process differs (see Inserts of Fig. 2). When the 500 nL are directly spotted on the plate, we observe a non-homogeneous distribution of the matrix (left insert) leading to an averaged intensity (n = 10) of 2085 (±876) for the Ang II signal on the MALDI-TOF MS spectra. On the contrary, the microfluidic droplet generator seems to play a concentrator role as we observe a high density of material in the location where the droplets are deposited on the plate (right insert). Consequently, the averaged intensity for the Ang II peak by MALDI-TOF analysis is equal to 59992 (±8871), approx. 30 fold higher than the one afforded by standard spotting method. This significant improvement of the sensitivity confirms the potential help of DMF to MALDI-TOF analysis.

The main difference observed between the two ways of spotting is the distribution of the crystallized matrix along the spot area. Indeed, the ratio matrix/surface is higher with the DMF-MALDI setup. In MALDI-TOF analysis, a more homogeneous sample distribution on the plate surface leads to more accurate, reproducible and sensitive analysis^12^. Homogeneity of both spotting processes was evaluated comparing the MS signal of Ang II obtained from 6 random locations within the same corresponding spot. The peak intensity of Ang II varies by more than 50% in conventional spotting (52%) whereas DMF spotting leads to a significant lower signal variation (12%) (illustration on Figure S0 in Supporting Information). The homogeneity of the dried material obtained with DMF spotting is an additional benefit for the analysis reliability.

The observed differences in spot size and material homogeneity might find their sources in the main parameters that differ in between DMF and conventional deposition such as i) the presence or not of oil, ii) the duration of deposition which is longer with DMF spotting compared to conventional spotting. When the deposition was performed with the microfluidic device in absence of oil, none MALDI-TOF MS signals were detected out from the chip (illustration on Figure S1 in Supporting Information) confirming the versatile oil role in DMF that prevents the well documented biofouling phenomena^25, 26^. Regarding MALDI-TOF MS signals intensity, the additive benefit of oil presence during spot drying process occurs but to a lesser extent when oil was included in conventional manual spotting. In these conditions, the Ang II MALDI-TOF MS signal was enhanced by a factor 2–4 when compared to conventional spotting without oil but not in a reproducible manner. The best signal increase was observed with the immediate oil addition on analyte mixture (illustration on Figure S2 in Supporting Information), longer delays were associated with weaker Ang II MALDI-TOF MS signals. Despite the signal enhancement observed, the signal improvement remains poor compared to the averaged one afforded from DMF spotting (30X). This difference may be due to manual conventional spotting restrictions characterized by one-shot deposition and delayed analyte surrounding by oil. In contrast, the digital microfluidic device delivers slowly on the MALDI plate the analyte surrounded by oil. The latter fact as well as the intimate surrounding of the analyte by oil within the microfluidic device may have a crucial impact for the analyte focusing phenomena observed on the plate and associated to peptide MALDI-TOF signals enhancement. One can postulate that the homogeneous, concentrated spot obtained on MALDI plate may result from an overall analyte recirculation due to hydrodynamics flows induced by Marangoni stress during the droplet evaporation^27, 28^. This recirculation phenomena is favored by DMF spotting that reduces the surface area contact between the sample and the plate and leads this way to a more localized crystallization. The main effect of DMF deposition method is then the improvement of the sensitivity by focused concentration and the enhancement of the crystallization homogeneity. Working range and detection limit of our set-up were first evaluated for Ang II MALDI-TOF MS detection.

**Figure 3:**
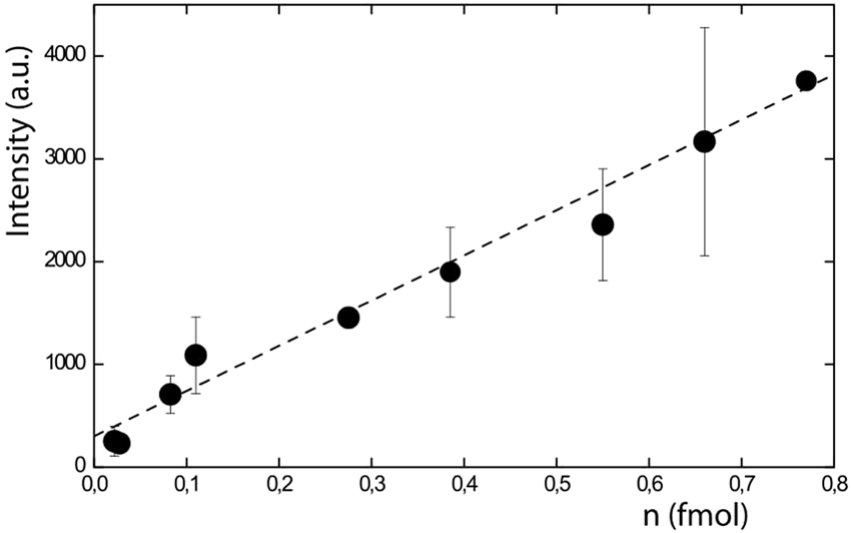
MALDI-TOF analysis of Ang II: Averaged intensity of the m/z = 1046.54 Da MALDI-TOF MS peak in function of the averaged calculated amount of Ang II spotted.

**Figure 4:**
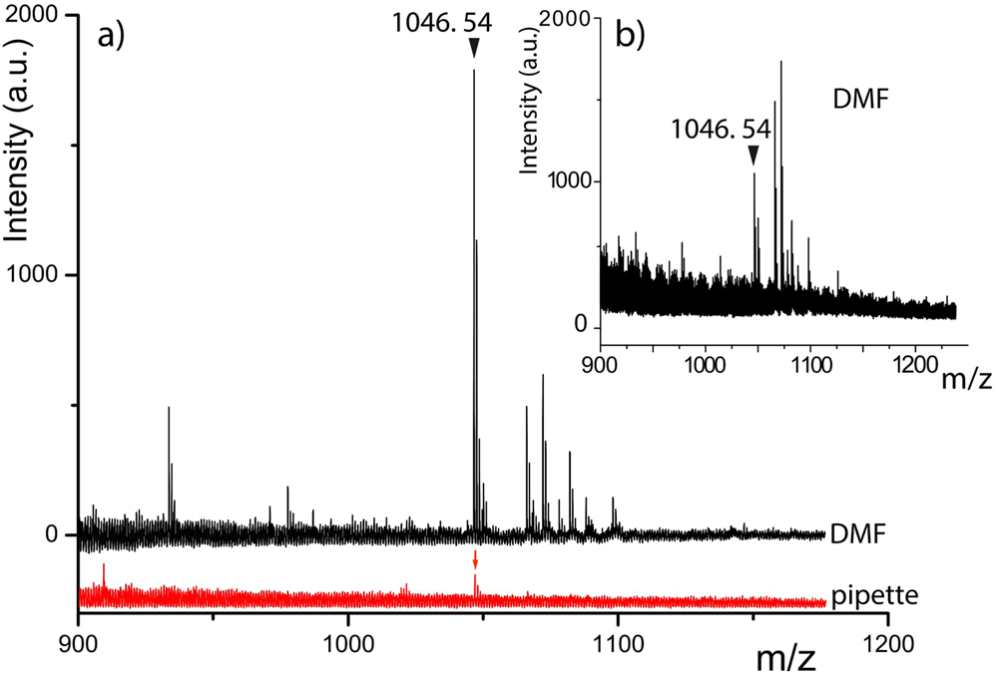
MALDI-TOF MS spectra of: (**a**) a 0.5 nM solution of Ang II spotted by DMF (black line) and standard pipette (red line shifted for clarity), and (**b**) DMF spotting of 0.1 nM solution of Ang II.

Each point on the curve of Fig. 3 corresponds to the averaged quantities of Ang II spotted in function of the averaged MS signal intensity of peak 1046.54 Da. We observe that the averaged MS signal intensity decreases with the amount of Ang II deposited on the spot (from 770 attomoles to 22 attomoles). The positive detection of such little quantity was achieved from solutions down to nanomolar level of Ang II i.e. ng/mL solution. The Ang II amounts spotted and the MALDI-TOF MS signals show a strong linear correlation which is a gained feature as MALDI-TOF MS signals are well known to be sample crystallization process dependent. Hence the reproducibility as well as the linearity in signal intensities are often difficult to obtain. For the sub-nanomolar solutions a proof of benefit of DMF method compared to conventional spotting method is illustrated in Fig. 4. It clearly shows the impact of our method on the detection sensitivity for a MALDI-TOF peptide analysis. Indeed when a 0.5 nM solution of Ang II is spotted, a quite intense peak corresponding to Ang II is detected (black line in 4a) from 50 attomoles spotted whereas the limit of detection with the standard method was reached from 500 attomoles deposited as a very weak signal is detected (red line in Fig. 4a). Spotting a 0.1 nM solution, detection of Ang II failed with conventional method, while on the contrary, DMF-MALDI method led to positive detection of Ang II from as few as 17 attomoles deposited (Fig. 4b). The results are very promising as 17 attomoles correspond to 9.3.10^6^ real molecules. The positive detection of such little quantity of Ang II by MALDI-TOF MS occurs thanks to a focusing effect of peptides within a tiny area, highlighting the high benefit of coupling digital microfluidic device with MALDI-TOF.

**Figure 5:**
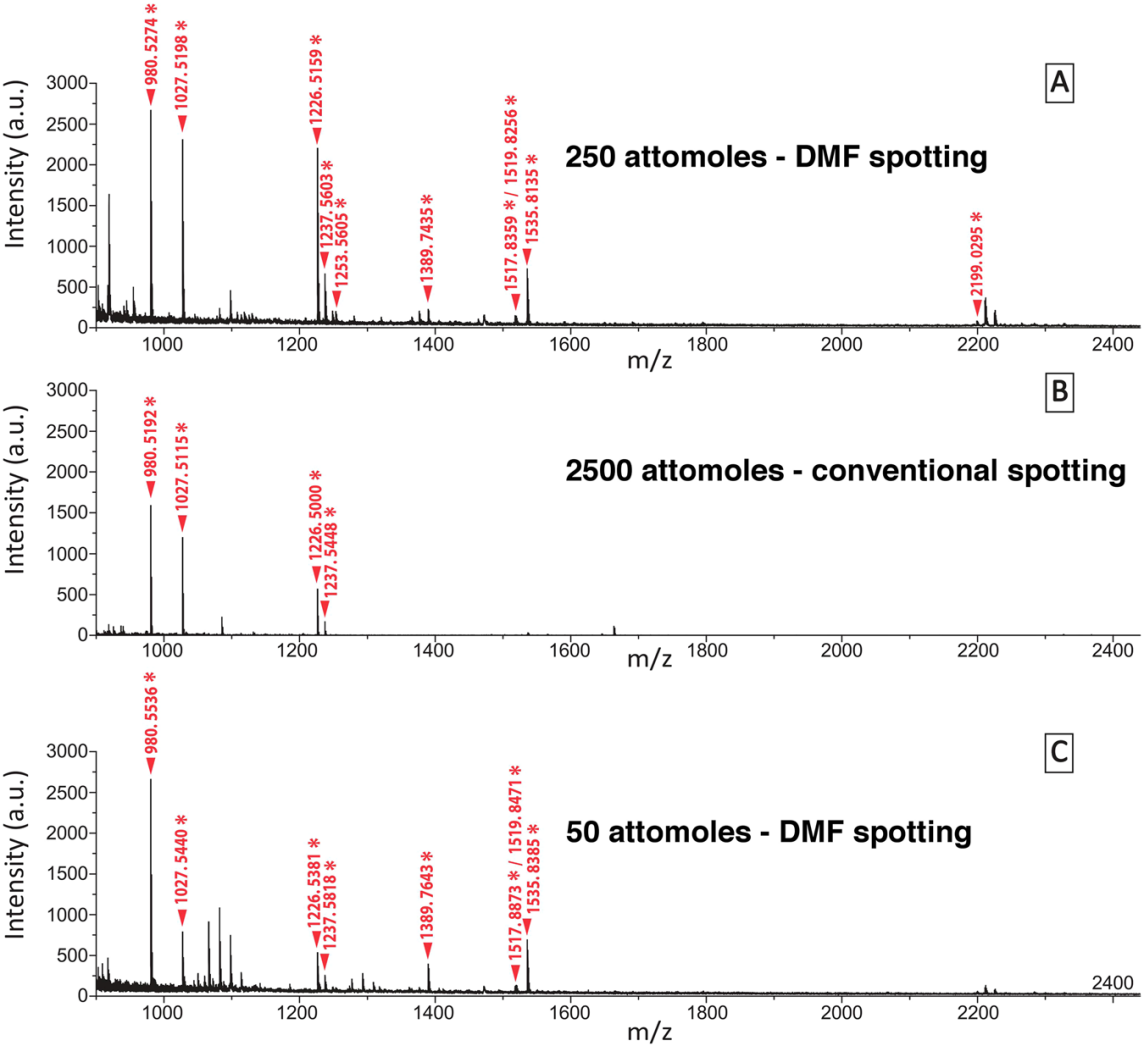
MALDI-TOF MS spectra of MMP12 digest: (**A**) DMF deposition of 250 attomoles, (**B**) Conventional pipette deposition of 2500 attomoles, (**C**) DMF deposition of 50 attomoles. The ticked masses and star-labeled peaks correspond to tryptic fragments of MMP12.

We then evaluated this concentration phenomena upon a mixture of peptides to assign the portability of our microfluidic device for proteomic era purposes. An extended benefit of the combination of our microfluidic device with MALDI-TOF MS detection as source for improvements of proteins analysis and identification was assigned using a mixture of tryptic fragments of a known metalloprotease (MMP12, Elastase, see amino acid sequence in Figure S3 in Supporting Information). This zinc metalloprotease belongs to a protease family which has been described to be involved in several pathological pathways^29^. The peptides mixture resulting from MMP12 digestion by trypsin was either directly spotted on MALDI plate using conventional protocol or spotted after passing through the digital microfluidic device. Starting from the same MMP12 digest solutions at respective concentration of 5 nM or 1 nM, conventional spotting method (0.5 *μ*L digest +0.5 *μ*L of matrix mixture) led to reference spots corresponding to 2.5 femtomoles and 500 attomoles whereas spots obtained from 100 nL (at 2.5 nM or 0.5 nM after the mix 1/1 with matrix solution) spotted out from the microfluidic device refers respectively to 250 attomoles and 50 attomoles. MALDI-TOF MS spectrum obtained from the 250 attomoles spot (Fig. 5, panel A) allowed the detection of 10 MMP12 fragments whereas only 4 were detected from the corresponding reference spot (2.5 femtomoles) (Fig. 5, panel B). MALDI-TOF MS spectra comparison unambiguously shows that studying the same sample using the digital microfluidic device increases the number of detected peaks relative to MMP12 fragments concomitantly with the intensities of most of the peaks (Fig. 5, panel A *vs* panel B). Thanks to the DMF spotting, these improvements could be obtained in a reproducible manner from several experiments (see the comparison of spotting methods in averaged signal intensities of 7 MMP12 fragments: Figure S4 in Supporting Information). MALDI-TOF MS spectrum obtained from the 50 attomoles spot also allowed the detection of 8 MMP12 peptide fragments (Fig. 5, panel C) whereas none exploitable peaks could be detected from conventional spotting of 500 attomoles. The latter observations had a direct impact on the corresponding Mascot protein score known that establish the significance of protein identification obtained from submission of mass list to protein databases. The submission of the MS data obtained from 250 attomoles and 50 attomoles DMF spots to databases led to valid Mascot identification of MMP12 (Mascot protein scores: respectively 102 and 85) whereas an unvalid protein score of 61 was attributed to MMP12 from MS data resulting from standard spot of 2.5 femtomoles of MMP12 digest and MMP12 was even not ranked in MASCOT from MS data obtained from conventional spotting of 500 attomoles of MMP12 digest (Table 1 and see Tables S1 and S2 in Supporting Information for a summary of detected fragments sequences and the theoretical expected m/z). Halved deposition duration of the 0.5 nM MMP12 digest solution out from the microfluidic device made possible to reach valid Mascot identification of MMP12 (Mascot protein scores: 82; number of peptides detected: 7 from a spot referring to 25 attomoles of MMP12 digest (see MALDI-TOF MS spectra in Figure S5 in Supporting Information). This demonstrated the high value of the combination of DMF and MALDI-TOF MS in terms of positive protein identification from spots containing protein digest in attomole range.

**Table 1:** Table summarizing quantities and results obtained for MMP12 identification with conventional or DMF deposition.

Starting MMP12 digest concentration*	5 nM		1 nM		
*deposition type*	*conventional*	*DMF*	*conventional*	*DMF*	
attomoles of digest on spot	2500	250	500	50	25
Number of MMP12 fragment detected	4	10	none	8	7
Mascot Mowse protein score	61	102	—	85	82
Protein rank	1	1	—	1	1
Identification significance	no < 66	**yes** > **66**	no < 66	**yes** > **66**	**yes** > **66**

*Before matrix addition.

## Conclusion

In this study we engineered a droplet based microfluidic (DMF) device dedicated to MALDI-TOF peptide analysis. We have shown the high value of DMF-MALDI combination that induces a concentration effect. This focusing of peptides within the tiny area on the MALDI plate leads to an enhanced peptide detection. This effect may be explained arising from both the oil presence and the specific way of DMF to deliver slowly the analyte on the plate. The detection sensitivity is improved (to few tens of attomoles) and positive detection can be achieved from sub-nanomolar peptides solutions. Enhanced peptide positive detection has a direct benefit for accurate protein identification. DMF-MALDI analysis offers promising potencies for easy and versatile treatment of protein samples. The field of proteomic analysis using MALDI-TOF spectrometry may take advantage of DMF-MALDI approach by integrating an enzymatic digestion step within the chip. Moreover, the beneficial impact of DMF combined to MALDI-TOF demonstrated here reinforces the interest of MALDI for broader applications afforded by DMF.

## Methods

### Materials and reagents

SU-8 photoresist (SU-8 3035) and propylene-glycol-monoether-acetate (PGMEA) developer were obtained from Microchem (Newton, MA, USA). Sylgard 184 (PDMS) prepolymer base and curing agent were purchased from Dow Corning (Midland, MI, USA). Silicon wafers were obtained from BT Electronics (France). Capillary tubes were purchased from CIL Cluzeau (France). Aquaphobe CF was purchased from Gelest (Morrisville, USA). Water was purified with an ultrapure system (Barnstead, IA, USA). The microfluidic set-up was made of a xy-stage (neMAXYS 200, Cetoni GmbH, Germany), a microfluidic flow control system MFCS-EZ (Fluigent, France), an Olympus CKX41 inverted microscope equipped with a pixeLINK CCD camera (pixeLINK, Canada), a DinoLite handheld USB camera (AM311-RO, AnMo Electronics Corporation, Hsinchu, Taiwan). The cleanroom falicities included an UV-lamp (KUB2, KLOE, France), a plasma cleaner (Harrick scientific, USA) and a light interferometer (Schaefer Technologies, GmbH). A 4800 spectrometer MALDI-TOF/TOF Proteomics Analyzer and Opti-TOF 384 well MALDI plate (Applied Biosystems, Foster City, USA, CA) were used for mass spectrometrey analysis of samples. Porcine trypsin (seq. grade modified, 20 *μ*g) for enzymatic digestion was from Promega. *α*-Cyano-4-hydroxy-cinnamic acid (HCCA) from Fluka was used as organic matrix for mass spectrometry analysis. Acetonitrile (ACN) was from Riedel-de Han. Sodium bicarbonate, Trifluoroacteic acid (TFA), trichloroacetic acid (TCA) were from SIGMA Aldrich. Oil (Perfluorohexane) was from Fluka. Commercial reagents were used without additional purification. Human MMP-12 with a mutation in position 171 (F171D) was produced in *E. coli* ^30^. Angiotensin II (Ang II, DRVYIHPF, MW 1045.54 Da) was purchased from Sigma-Aldrich.

### Fabrication of microfluidic chip

The microdevice was fabricated in two main steps: master fabrication and replica molding. Master fabrication was performed using the standard soft lithography method^31^. The microstructures (channels and restrictions) were designed in a computer aided design program. Transparencies of the CAD-generated patterns were then created using commercial services. The SU-8 layer was obtained by spin coating the negative SU-8 3035 photoresist (viscosity 7.5 cSt) at 300 rpm for 10 s and 1000 rpm for 30 s. The wafer was soft baked for 30 min at 95 °*C*. After being cooled down, the wafer was aligned with the transparency pattern and insulated (365 nm, 17 s at 100% energy) with a UV lamp. The wafer was developed in the appropriate solvent and the height of the SU-8 structure (95 *μ*m) was measured with a light interferometer. For the replica molding, the polydimethylsiloxane Sylgard 184 elastomer and the curing agent were mixed in 10:1 (w/w). The mixture was poured on the master and put in vacuum chamber to remove air bubbles before being heated in oven at 60 °*C* for few hours to cure the PDMS. Holes (300 *μ*m diameter) were punched for the inlets and the PDMS replicates were sealed to glass slides following oxygen plasma treatment at 200 mTorr for 1 min using a plasma cleaner.

### Surface treatment

The inner walls of the microchannels were treated with Aquaphobe CF. Immediately after the plasma treatment, the channels were filled with a coating solution composed of 2% Aquaphobe CF in toluene. After 5 minutes incubation, the channels were rinsed extensively with water and cleared out using N_2_ gun to remove any liquid from the channels. The chip was heated at 110 °*C* for 20 minutes on a hot plate before being transfered to the 90 °*C* dry oven overnight. Once ready, the chip was kept in a vacuum storage box before use.

### Liquid management

A microfluidic flow control system was used to fill the channels with both aqueous and oil phase. Perfluorohexane was used as fluid carrier. Fluorinated alcohol was added to the oil phase at 10% (w/w) to improve the wettability of the oil phase towards the channel walls. PEEK capillaries (510 *μ*m × 125 *μ*m) and HPFA+ (360 *μ*m × 100 *μ*m) were used as inlet and outlet tubing respectively. Droplets were created in a T-junction inside the microchip. An inverted microscope equipped with a CCD camera was used to monitor droplet transport inside the chip microchannels. The commercial MALDI plate was mounted on a xy-stage with a worktable of 200 mm × 200 mm. These travel ranges enabled the use of the complete MALDI plate format for droplet deposition. The HPFA+ outlet tube ending was set on the z-stage of the nemAXYS for the droplet deposition on the MALDI plate. Additionally, a USB camera was used to monitor the spotting process. A high precision mechanical alignment of the MALDI plate spotting area with the outlet tube extremity was made possible by an accurate programming of the xy-stage software. Each MALDI plate spots coordinates were calculated in order to easily adjust the outlet tube nozzle with any spot.

### Samples and matrix preparations

For each series of experiments, fresh matrix and fresh peptides or proteins samples were prepared. Matrix solutions were prepared using HCCA diluted at 2 mg/mL in a mixture of acidified H_2_O/ACN (75:25 v/v). The preparation was vortexed for 5 minutes and sonicated to complete solubilization. Optimized HCCA solution used 0.1% TFA or 1% TCA for reference conventional spotting and TCA for DMF spotting. Solution of Angiotensin II were prepared as follow: aliquots at 100 fmol/*μ*L in H_2_O/ACN (80:20 v/v) and 0.01% TFA were diluted by adding the corresponding volume of a mixture H_2_O/ACN (75:25 v/v) and the resulting solution was mixed (1:1 v/v) with matrix solution to reach 0.1 nM, 0.5 nM, 1 nM, 5 nM, 10 nM and 20 nM. Conventional (or standard) pipetting deposition (or spotting) and DMF spotting were performed from same starting peptide solutions mixed (1:1 v/v) with matrix. Detection sensitivity test was performed by depositing and analyzing decreasing amounts of Ang II on a MALDI plate. Solutions containing Ang II at different concentrations were used to generate droplets through the device to afford on the MALDI plate spots (10 replicates per concentration) of Ang II different amounts. The latter were calculated from the counting of the droplet numbers (monitored under camera) and the set droplet constant volume (4 nL). Once the spots analyzed, the quantities of Ang II spotted were related to the corresponding MALDI-TOF MS signal intensities. In the particular case where oil was tested for conventional spotting, 1 *μ*L of Ang II (5 nM) and matrix 75:25 (H_2_O/ACN) with 0.5% of TCA in the final mixture were used with or without oil. For control experiments using continuous flow through the microfluidic device in absence of oil, volumes of 200 to 1000 nL of Ang II (5 nM) and matrix 75:25 (H_2_O/ACN) with 0.5% of TCA in the final mixture were injected. Samples of peptides mixture at chosen concentration were prepared from the enzymatic digestion of MMP12 by porcine trypsin that produces MMP12 fragments. Stock solution of peptide fragments (MMP12 digest) was prepared for each experiments as follow: 3.4 picomoles of MMP12 diluted in 16 *μ*L of 50 mM sodium bicarbonate/acetonitrile solution (9:1) were mixed with 1 *μ*L of freshly prepared porcine trypsin solution (100 ng/*μ*L in a mixture of 50 mM sodium bicarbonate/acetonitrile (9:1)). Sample at 0.2 picomole of MMP12 per *μ*L was heated at 50 °*C* for 2 hours under gentle shaking. The stock sample containing MMP12 tryptic fragments was diluted in H_2_O/ACN (75:25 v/v) to afford samples at 5 nM and 1 nM before mixing with matrix solution (ratio 1/1). Conventional spotting of peptides mixture (MMP12 digest) was performed using 0.5 *μ*L of MMP12 digest sample spotted sequentially with 0.5 *μ*L matrix solution containing optimized HCCA quantity per mL (2 mg) in H_2_O/ACN and TFA 0.1%. In optimized DMF spotting, peptides mixture solutions were mixed with an equal volume of matrix solution with optimal composition (HCCA 2 mg/mL in H_2_O/ACN (75:25 v/v, TCA 1%) before the droplet generation process and the spotting. Once can note that a particular attention has been paid to the H_2_O/ACN mixture ratio and the dilution process of protein or peptide sample. Usually, matrix solution (composed of H_2_O/ACN (1:1 v/v) with a low percentage of acid) is diluted (1:1) with an aqueous solution containing the analyte prior the analysis, resulting in 25% of ACN in the final composition. Therefore, the ratio for the preparation of all solutions (matrix + sample) in all the DMF experiments presented in this paper is 75:25 (H_2_O/ACN) with 0.5% of TCA in the final mixture.

### PMF MALDI-TOF analysis

The DataExplorer software (Ver 4.9) from ABI was used to generate ASCII peak lists for peptide mass fingerprinting (PMF) MS analyses. Each peak list containing m/z greater than 900 was manually applied for searches using MASCOT software (www.matrixscience.com) in the NCBInr database. The parameters used for the search were as follows: a taxonomy restriction was placed to Homo sapiens (human), one missed trypsin cleavage was allowed, a maximum mass tolerance was set at 50 ppm and methionine and HW oxidations were set as a variable modification. MASCOT protein hit Mowse scores greater than 66 (assuming p < 0.05) were considered significant for MMP12.

## Electronic supplementary material

Supplementary Information

## References

[1] Hajduk J, Matysiak J, Kokot ZJ (2016). Challenges in biomarker discovery with maldi-tof {MS}. Clinica Chimica Acta.

[2] Calligaris, D., Villard, C. & Lafitte, D. Advances in top-down proteomics for disease biomarker discovery. *Journal of Proteomics* Mass Spectrometry - One of the Pillars of Proteomics **74**, 920–934 (2011). 10.1016/j.jprot.2011.03.030 21477672

[3] Chao T-C, Hansmeier N, Halden RU (2010). Towards proteome standards: the use of absolute quantitation in high-throughput biomarker discovery. Journal of proteomics.

[4] Petricoin EF, Zoon KC, Kohn EC, Barrett JC, Liotta LA (2002). Clinical proteomics: translating benchside promise into bedside reality. Nature reviews Drug discovery.

[5] Wilen, C. B., McMullen, A. R. & Burnham, C.-A. D. Comparison of sample preparation, instrumentation platforms, and contemporary commercial databases for maldi-tof ms identification of clinically relevant mycobacteria. *Journal of clinical microbiology* JCM–00567 (2015). 10.1128/JCM.00567-15 PMC4473241 25972426

[6] Martin-Lorenzo M (2014). 30 *μ*m spatial resolution protein maldi msi: In-depth comparison of five sample preparation protocols applied to human healthy and atherosclerotic arteries. Journal of proteomics.

[7] Wiangnon K, Cramer R (2015). Sample preparation: a crucial factor for the analytical performance of rationally designed maldi matrices. Analytical chemistry.

[8] Kim YE, Yi SY, Lee C-S, Jung Y, Chung BH (2012). Gold patterned biochips for on-chip immuno-maldi-tof ms: Spr imaging coupled multi-protein ms analysis. Analyst.

[9] Longobardi S (2015). A simple maldi plate functionalization by vmh2 hydrophobin for serial multi-enzymatic protein digestions. Analytical and bioanalytical chemistry.

[10] Dunn JD, Igrisan EA, Palumbo AM, Reid GE, Bruening ML (2008). Phosphopeptide enrichment using maldi plates modified with high-capacity polymer brushes. Analytical chemistry.

[11] Pabst M (2013). Self-aliquoting microarray plates for accurate quantitative matrix-assisted laser desorption/ionization mass spectrometry. Analytical Chemistry.

[12] Kudina O, Eral B, Mugele F (2016). e-maldi: an electrowetting-enhanced drop drying method for maldi mass spectrometry. Analytical chemistry.

[13] Mazutis L (2013). Single-cell analysis and sorting using droplet-based microfluidics. Nature protocols.

[14] Mary P (2011). Analysis of gene expression at the single-cell level using microdroplet-based microfluidic technology. Biomicrofluidics.

[15] Nightingale, A. M., Phillips, T. W., Bannock, J. H. & de Mello, J. C. Controlled multistep synthesis in a three-phase droplet reactor. *Nature communications* **5** (2014). 10.1038/ncomms4777 PMC4024758 24797034

[16] Nahavandi S (2014). Microfluidic platforms for biomarker analysis. Lab on a Chip.

[17] He M (2005). Selective encapsulation of single cells and subcellular organelles into picoliter-and femtoliter-volume droplets. Analytical chemistry.

[18] Yobas L, Martens S, Ong W-L, Ranganathan N (2006). High-performance flow-focusing geometry for spontaneous generation of monodispersed droplets. Lab Chip.

[19] Marz A, Henkel T, Cialla D, Schmitt M, Popp J (2011). Droplet formation via flow-through microdevices in raman and surface enhanced raman spectroscopy-concepts and applications. Lab Chip.

[20] Edgar JS (2006). Capillary electrophoresis separation in the presence of an immiscible boundary for droplet analysis. Analytical chemistry.

[21] Koster S, Verpoorte E (2007). A decade of microfluidic analysis coupled with electrospray mass spectrometry: An overview. Lab on a Chip.

[22] Hatakeyama T, Chen DL, Ismagilov RF (2006). Microgram-scale testing of reaction conditions in solution using nanoliter plugs in microfluidics with detection by maldi-ms. Journal of the American Chemical Society.

[23] Momotenko D (2012). Electrochemical push–pull scanner with mass spectrometry detection. Analytical chemistry.

[24] Küster SK (2013). Interfacing droplet microfluidics with matrix-assisted laser desorption/ionization mass spectrometry: label-free content analysis of single droplets. Analytical chemistry.

[25] Flemming H (2002). Biofouling in water systems - cases, causes and countermeasures. Applied Microbiology and Biotechnology.

[26] Wong I, Ho C-M (2009). Surface molecular property modifications for poly(dimethylsiloxane) (PDMS) based microfluidic devices. Microfluidics and Nanofluidics.

[27] Deegan R (1997). Capillary flow as the cause of ring stains from dried liquid drops. Nature.

[28] Tsoumpas Y, Dehaeck S, Rednikov A, Colinet P (2015). Effect of Marangoni Flows on the Shape of Thin Sessile Droplets Evaporating into Air. Langmuir.

[29] Kessenbrock K, Plaks V, Werb Z (2010). Matrix metalloproteinases: regulators of the tumor microenvironment. Cell.

[30] Banci L (2003). Expression and high yield production of the catalytic domain of matrix metalloproteinase 12 and of an active mutant with increased solubility. Journal of Molecular Catalysis a-Chemical.

[31] Sia S, Whitesides G (2003). Microfluidic devices fabricated in poly(dimethylsiloxane) for biological studies. Electrophoresis.

