# Supplementary material for: DMF-MALDI: droplet based microfluidic combined to MALDI-TOF for focused peptide detection
Source: Sci Rep. 2017 Jul 28;7:6756. doi: 10.1038/s41598-017-06660-8 (PMC5533719; doi:10.1038/s41598-017-06660-8)
Supplement: Supplementary file 1 — Supplementary Information [file 41598_2017_6660_MOESM1_ESM.pdf]

Supporting Information

DMF-MALDI: droplet based microfluidic combined to MALDI-TOF for focused peptide detection

Kiarach Mesbah,[1] Robert Thai,[2] Sarah Bregant,[2,*] and Florent Malloggi[1,*]

LIONS, NIMBE, CEA, CNRS, Université Paris-Saclay, CEA Saclay 91191 Gif sur Yvette Cedex, France.
SIMOPRO, JOLIOT, DRF, CEA, Université Paris-Saclay, CEA Saclay 91191 Gif sur Yvette Cedex, France.

E-mail: florent.malloggi@cea.fr ; sarah.bregant@cea.fr

Supporting Information includes mainly complementary results of the MMP12 digest study by DMF-MALDI.

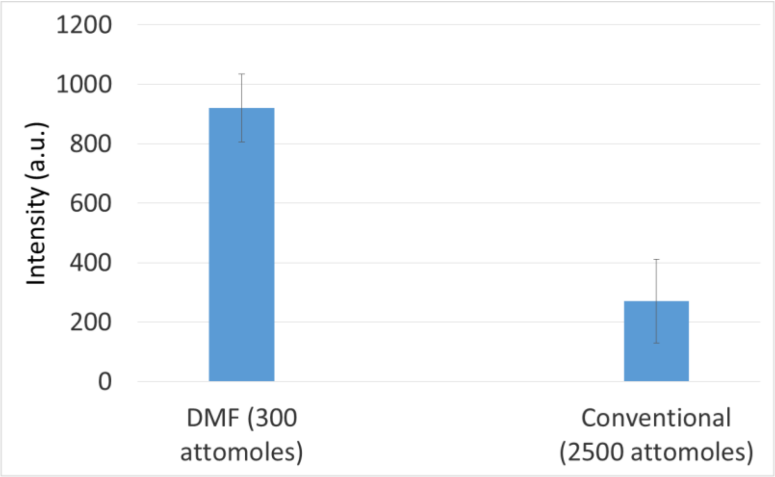

Figure S0. MALDI-TOF MS signal variation for m/z = 1046.54 Da Angiotensin II peak on 6 random locations within the same DMF spotted area or conventionally spotted area. Variation of 12% was obtained from DMF spotting of 300 attomoles and variation of 52% was obtained from conventional spotted of 2500 attomoles of Angiotensin II.

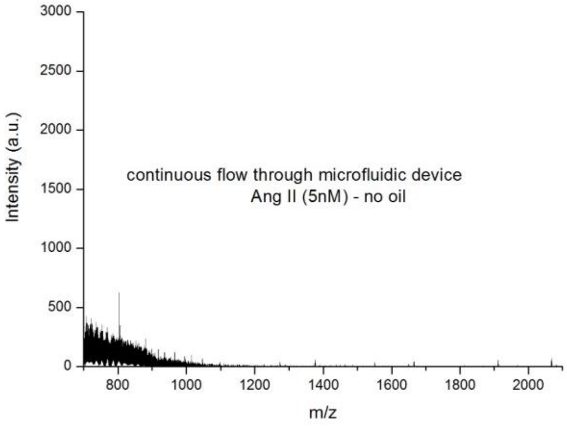

Figure S1. Example of MALDI-TOF MS spectra: Ang II matrix mixture 5nM injected directly in the PDMS chip without oil.

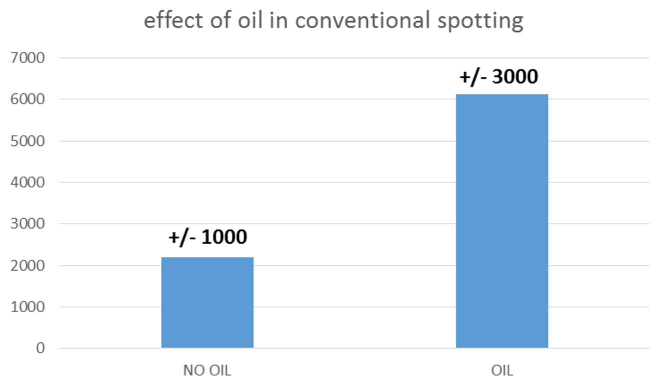

Figure S2. Influence of oil addition for the spotting. MALDI-TOF MS signal variation for m/z = 1046.54 Da Angiotensin II peak using conventional method deposition including oil or not in the protocol.

MGPVWRKHYITYRINNYTPDMNREDVDYAIRKAFQVWSNVTPLKFSKINTGMADILVVFARGAHGDD
HAFDGKGGILAHAFGPGSGIGGDAHFDEDEFWTTHSGGTNLFLTAVHEIGHSLGLGHSSDPKAVMFPTY
KYVDINTFRLSADDIRGIQSLYG

Figure S3. MMP12 amino-acids sequence

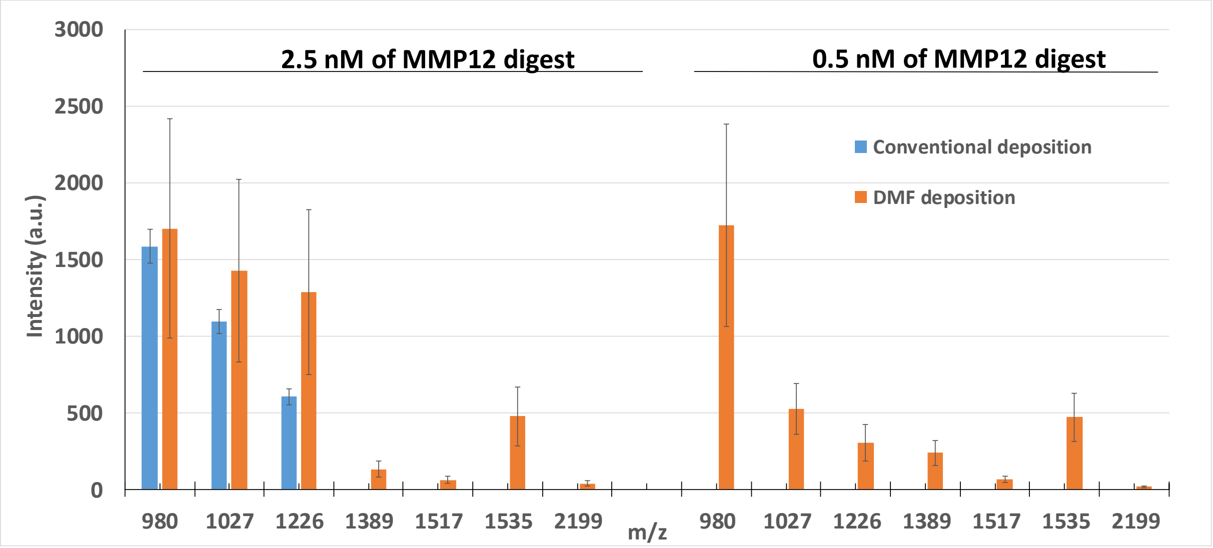

Figure S4. Averaged intensities of MALDI-TOF MS peaks referring to 7 MMP12 fragments. Conventional spotting (blue bars) and DMF spotting (orange bars) of 2.5nM (left and 0.5nM (right) MMP12 digest solution.

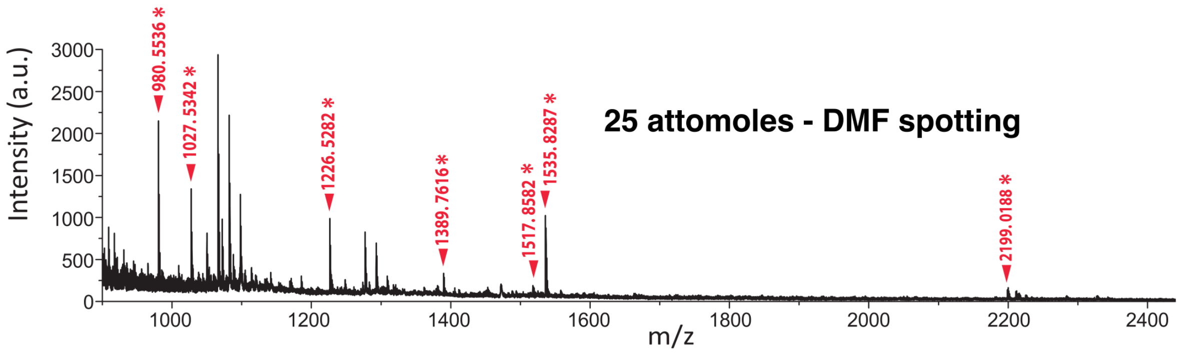

Figure S5 - MALDI-TOF MS spectra of 25 attomoles of MMP12 digest - DMF spotting

Starting MMP12 digest concentration	5 nM		1 nM		
deposition type	*conventional*	*DMF*	*conventional*	*DMF*	
attomoles of digest on spot	2500	250	500	50	25
MMP12 fragment detected	4	10	none	8	7
Valid identification	no	√	no	√	√
Sequence detected	***m/z detected (MH⁺) - Da***				
KHYITYR	980,5192	980,5274	-	980,5536	980,5429
YVDINTFR	1027,5115	1027,5198	-	1027,5440	1027,5342
GAHGDDHAFDGK	1226,5000	1226,5159	-	1226,5381	1226,5282
INNYTPDMNR	1237,5448	1237,5603	-	1237,5818	1389,7616
INNYTPDMNR+Ox	-	1253,5605	-	-	-
AFQVWSNVTPLK	-	1389,7435	-	1389,7643	-
KAFQVWSNVTPLK	-	1517,8359	-	1517,8873	1517,8582
INTGMADILVVFAR	-	1519,8256	-	1519,8471	-
INTGMADILVVFAR+Ox	-	1535,8135	-	1535,8385	1535,8287
INNYTPDMNREDVDYAIR	-	2199,0195	-	-	2199,0188

Table S1- Summary of m/z detected and corresponding MMP12 fragment sequence for experiments implying conventional and DMF spotting from starting digest solution 5 nM and 1 nM.

Fragments sequence	***Theoretical expected m/z (MH⁺) - Da***
KHYITYR	980,5312
YVDINTFR	1027,5207
GAHGDDHAFDGK	1226,5184
INNYTPDMNR	1237,5630
INNYTPDMNR+Ox	1253,5579
AFQVWSNVTPLK	1389,7525
KAFQVWSNVTPLK	1517,8474
INTGMADILVVFAR	1519,8301
INTGMADILVVFAR+Ox	1535,8250
INNYTPDMNREDVDYAIR	2199,0135

Table S2 - Summary of theoretical expected m/z for the MMP12 fragments observed
